# Supplementary material for: Synthesis and Properties of Magnetic Aryl-Imidazolium Ionic Liquids with Dual Brønsted/Lewis Acidity
Source: Materials (Basel). 2018 Dec 13;11(12):2539. doi: 10.3390/ma11122539 (PMC6316537; doi:10.3390/ma11122539)
Supplement: Supplementary file 1 [file materials-11-02539-s001.pdf]

materials
MDPI

Article

Synthesis and Properties of Magnetic Aryl-Imidazolium Ionic Liquids with Dual Brønsted/Lewis Acidity

Jui-Cheng Chang [1,2,*,†], Che-Hsuan Yang [3,†], I-Wen Sun [3], Wen-Yueh Ho [4,*] and Tzi-Yi Wu [1]

1. Department of Chemical and Materials Engineering, National Yunlin University of Science and Technology, Yunlin 64002, Taiwan; wuty@yuntech.edu.tw
2. Bachelor Program in Interdisciplinary Studies, National Yunlin University of Science and Technology, Yunlin 64002, Taiwan
3. Department of Chemistry, National Cheng Kung University, Tainan 70101, Taiwan; yangvul3rm03@gmail.com (C.-H.Y.); iwsun@mail.ncku.edu.tw (I.-W.S.)
4. Department of Cosmetic Science, Chia Nan University of Pharmacy & Science, Tainan 71710, Taiwan

* Correspondence: changjui@yuntech.edu.tw (J.-C.C.); rickho@mail.cnu.edu.tw (W.-Y.H.); Tel.: +886-5-534-2601 (ext. 4685) (J.-C.C.); +886-6-266-4911 (ext. 2418) (W.-Y.H.); Fax: +886-5-531-2071 (J.-C.C.); +886-6-266-7324 (W.-Y.H.)

† These authors contributed equally to this work

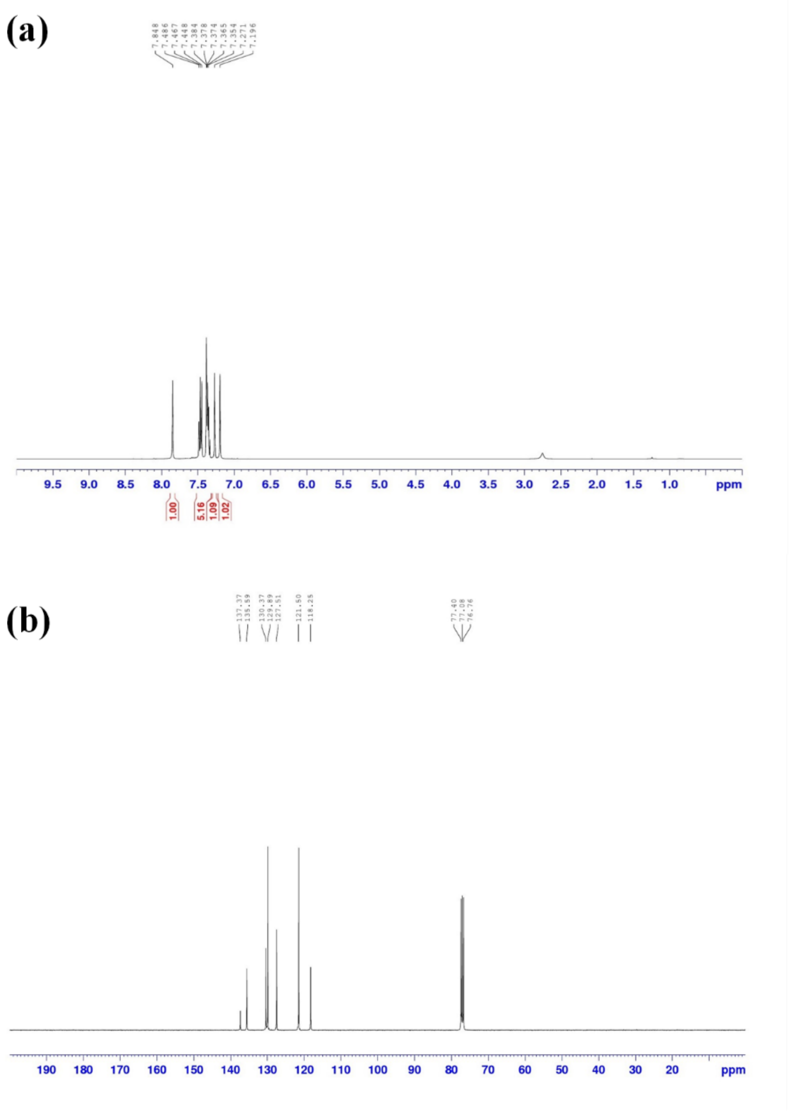

Figure S1a. (**a**) ^{1}H and (**b**) ^{13}C NMR spectra of 3a.

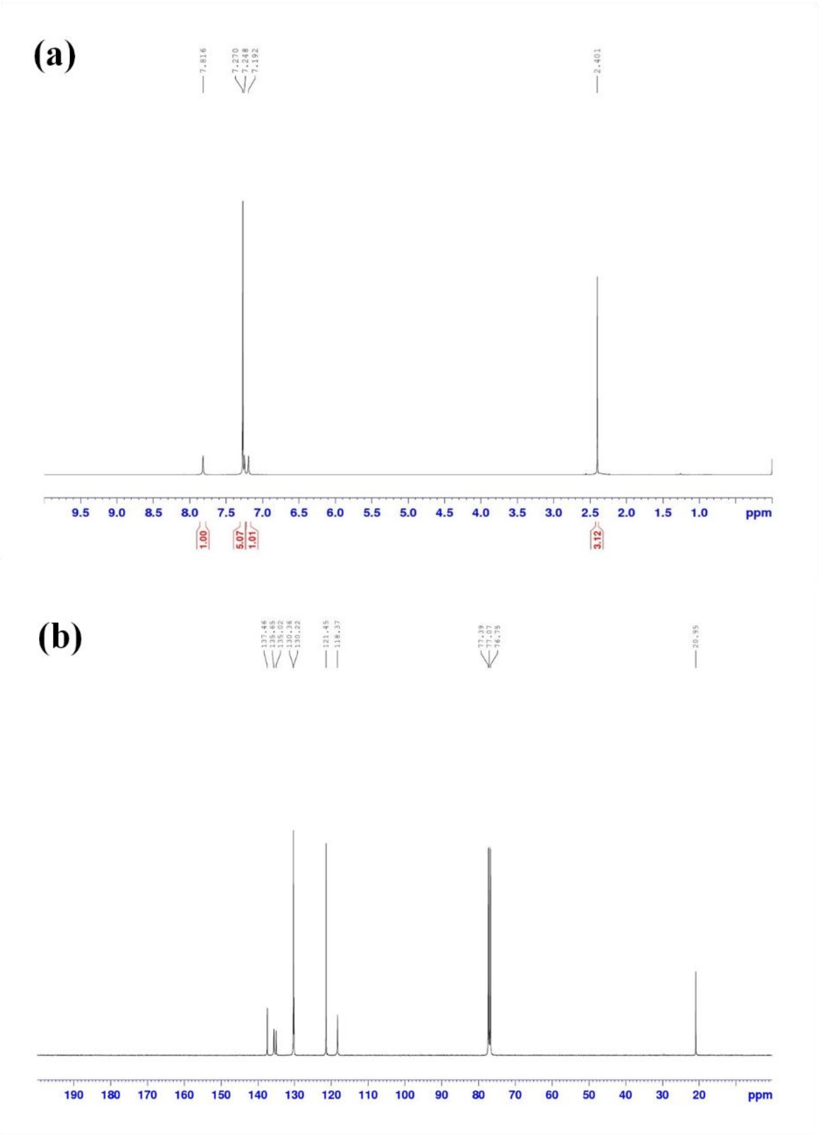

Figure S1b. (**a**) ^{1}H and (**b**) ^{13}C NMR spectra of 3b.

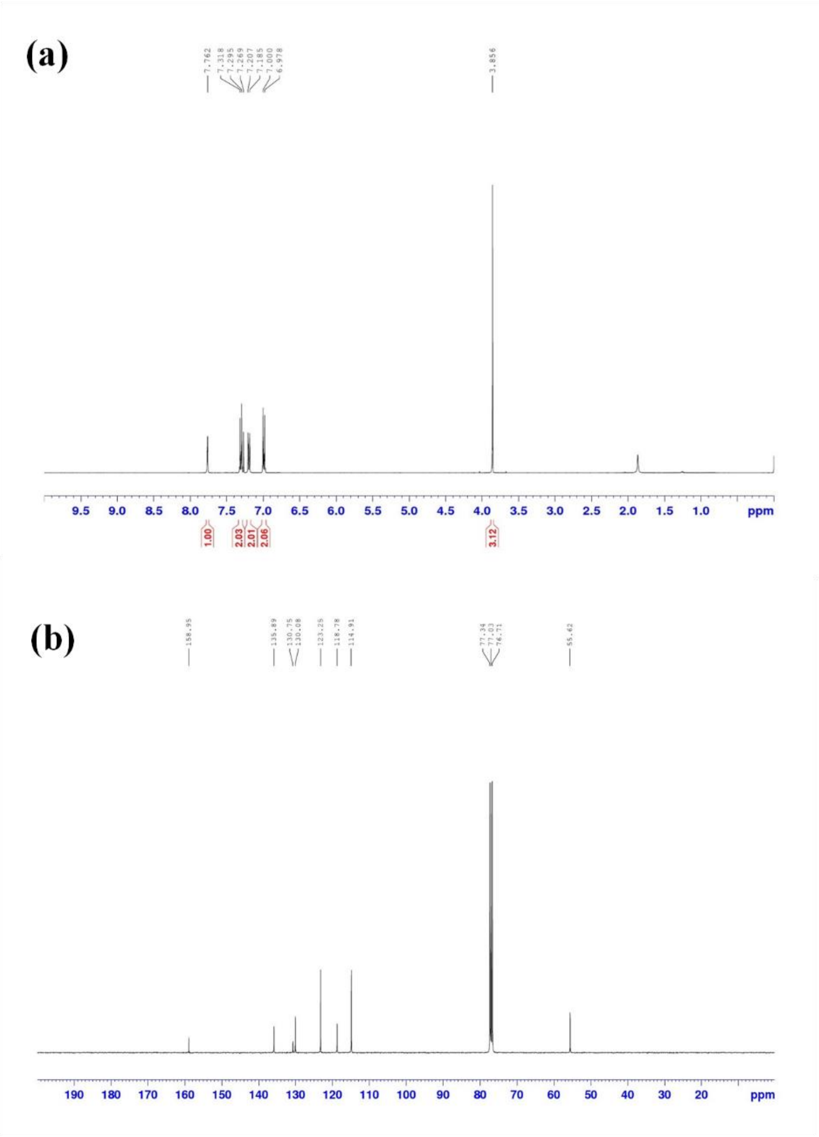

Figure S1c. (**a**) ^{1}H and (**b**) ^{13}C NMR spectra of 3c.

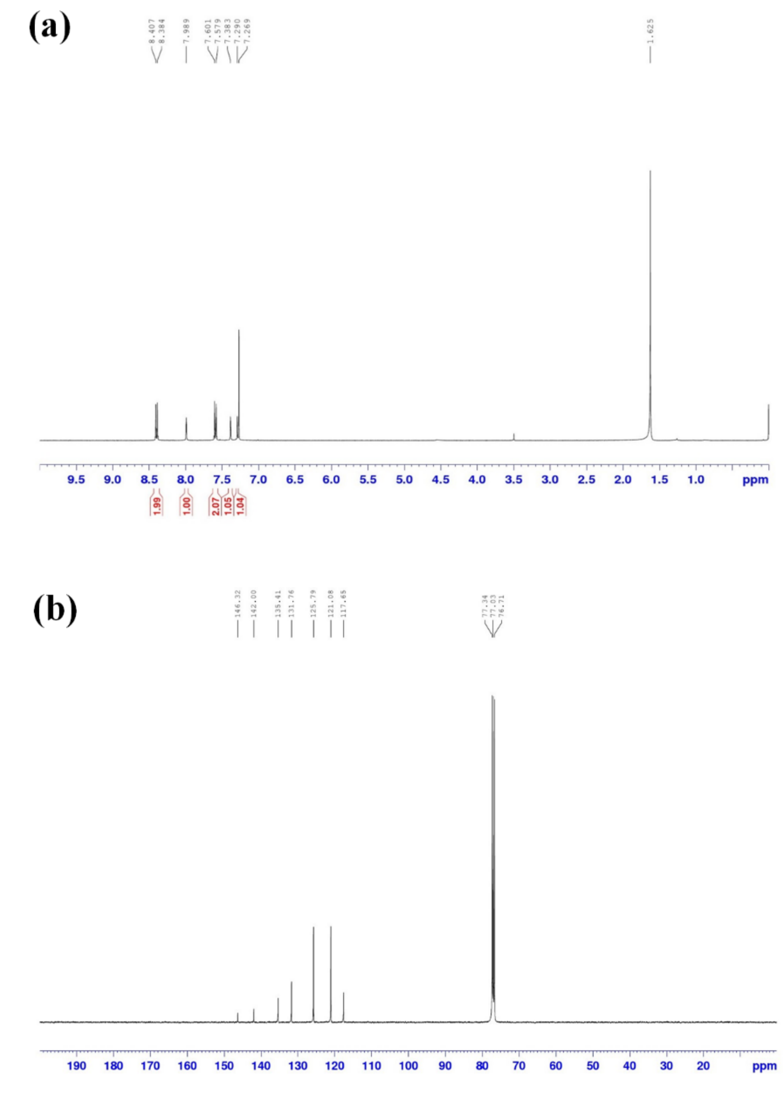

Figure S1d. (**a**) ^{1}H and (**b**) ^{13}C NMR spectra of 3d.

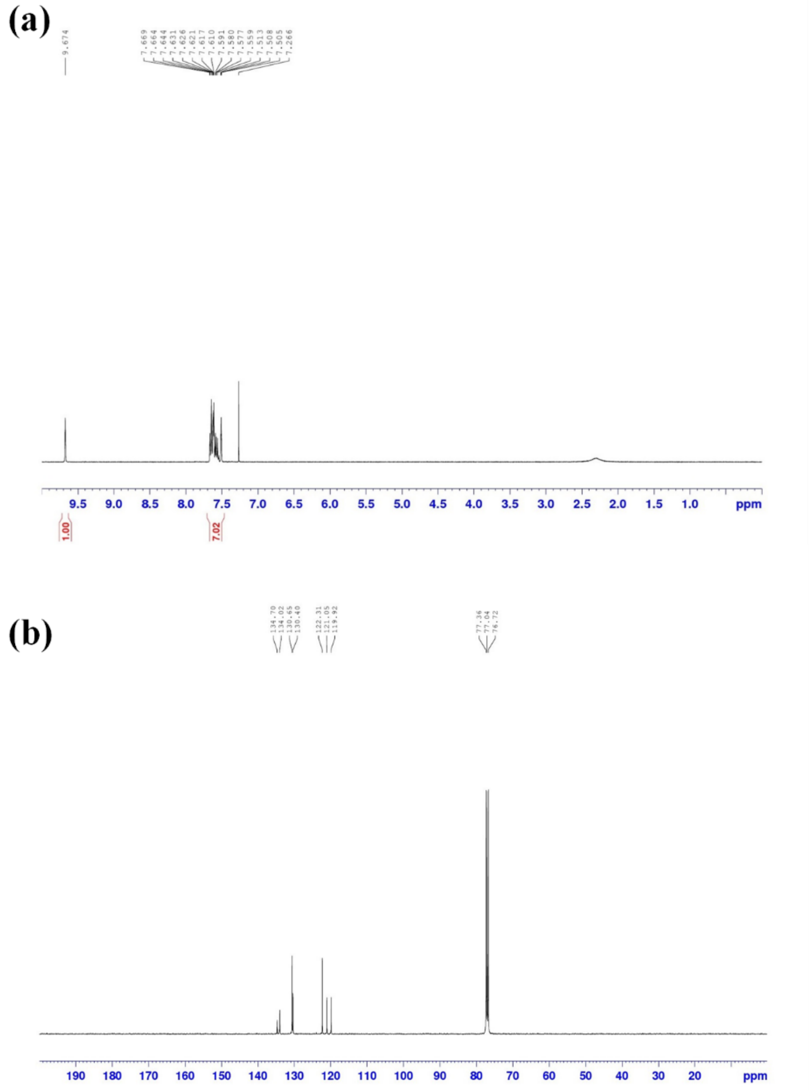

Figure S1e. (**a**) ^{1}H and (**b**) ^{13}C NMR spectra of 4a.

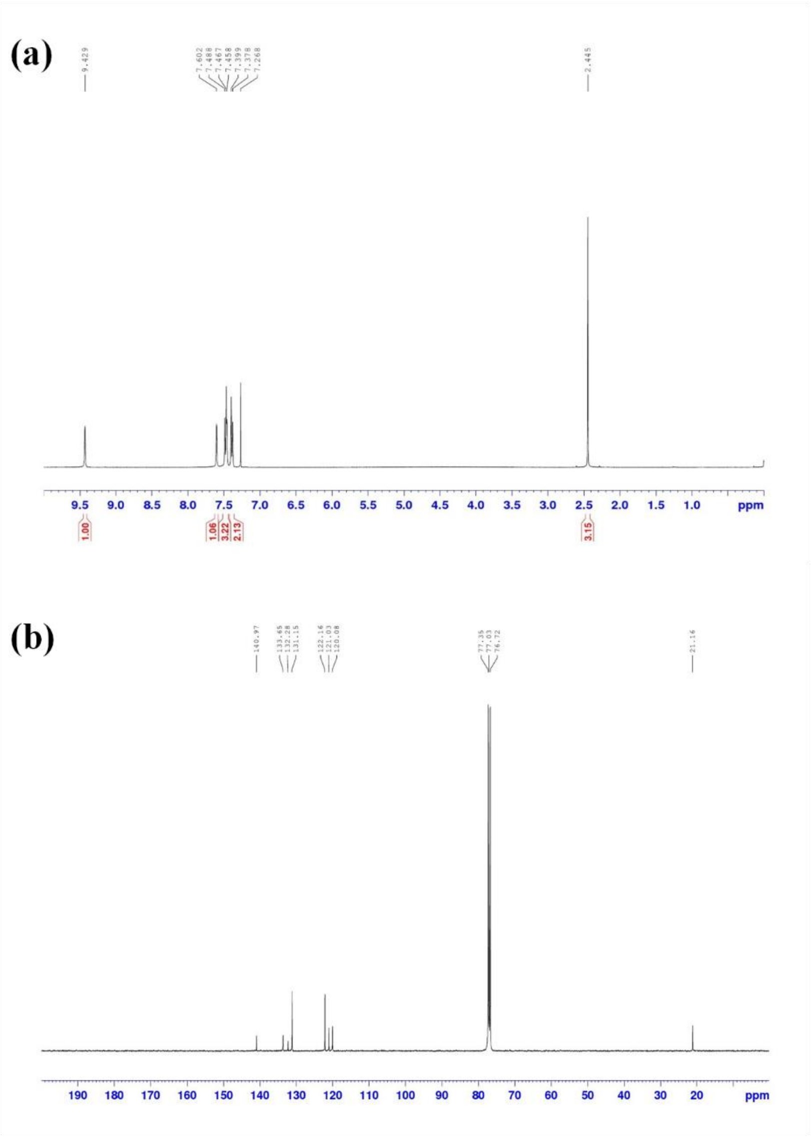

Figure S1f. (**a**) ^{1}H and (**b**) ^{13}C NMR spectra of 4b.

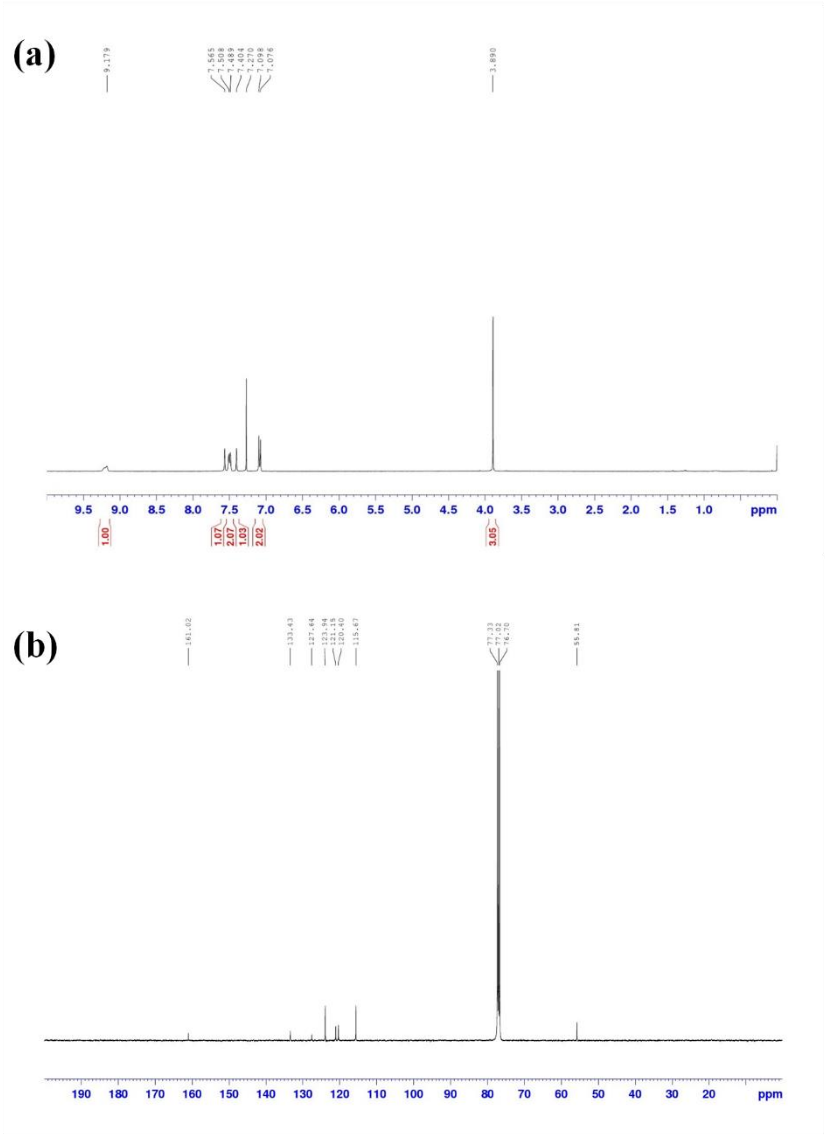

Figure S1g. (**a**) ^{1}H and (**b**) ^{13}C NMR spectra of 4c.

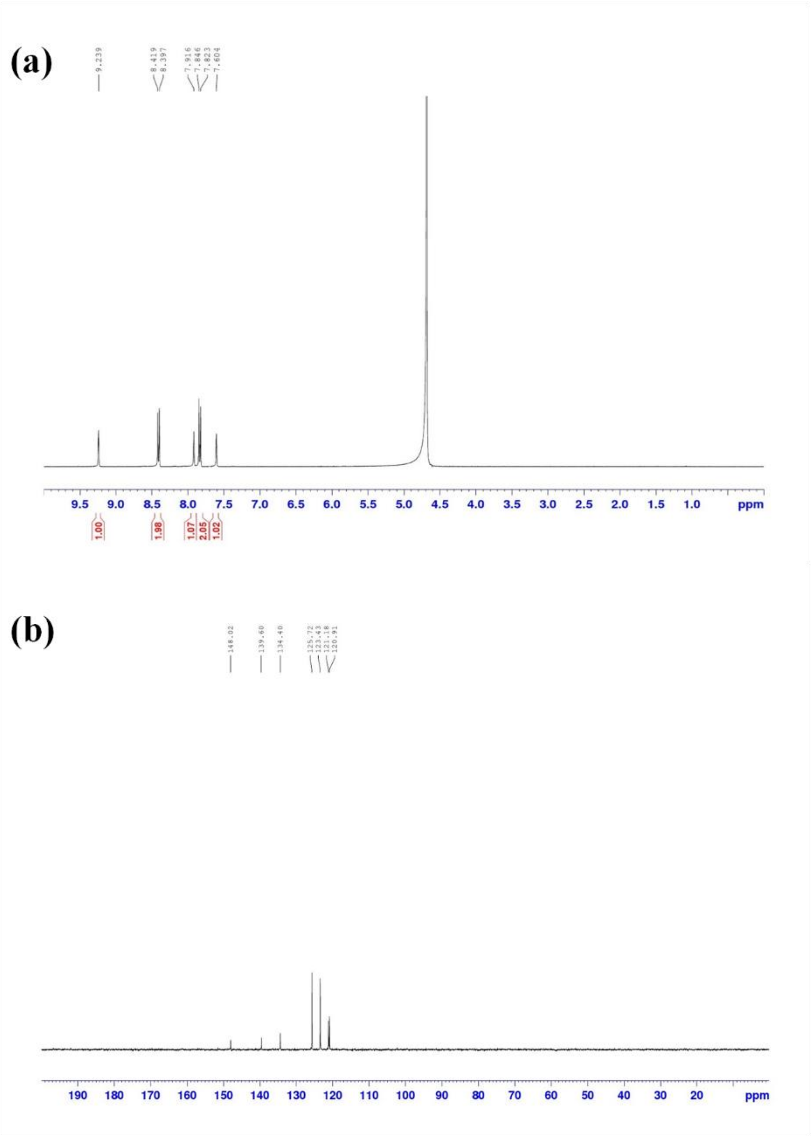

Figure S1h. (**a**) ^{1}H and (**b**) ^{13}C NMR spectra of 4d.

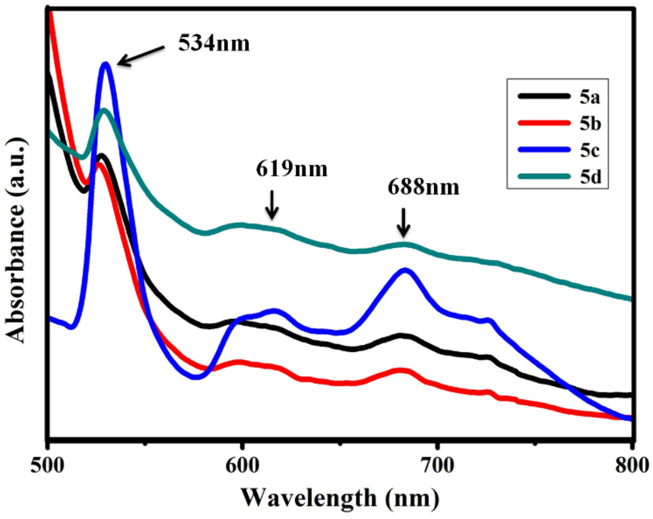

Figure S2. UV-Vis absorption spectra of B-L MILs 5a–5d (solvent: CH_3CN).

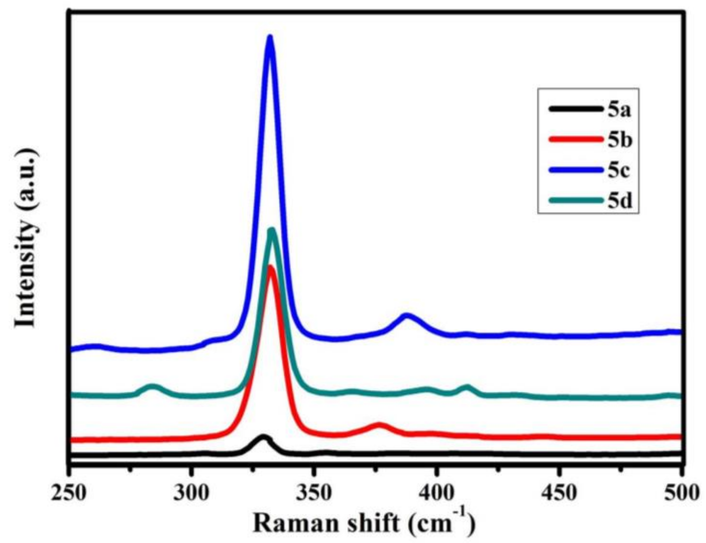

Figure S3. Raman spectra of B-L MILs 5a–5d.

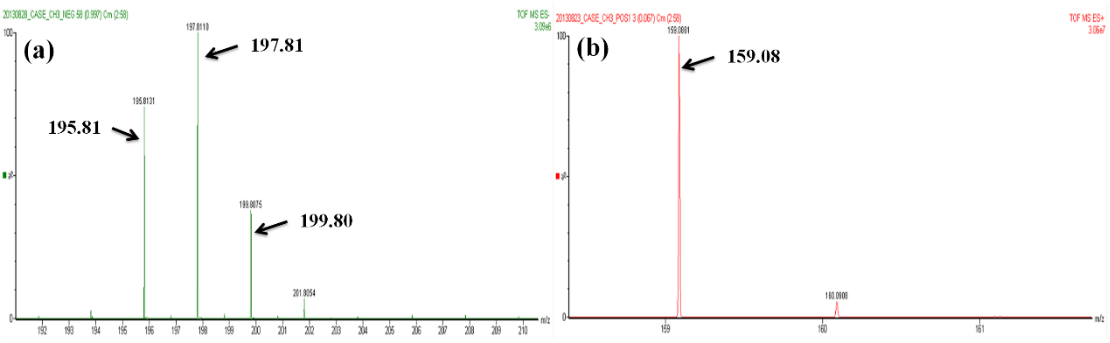

Figure S4a. Mass spectra (ionization source: electrospray ionization) of B-L MIL 5b. (**a**) Anion and (**b**) cation fragments.

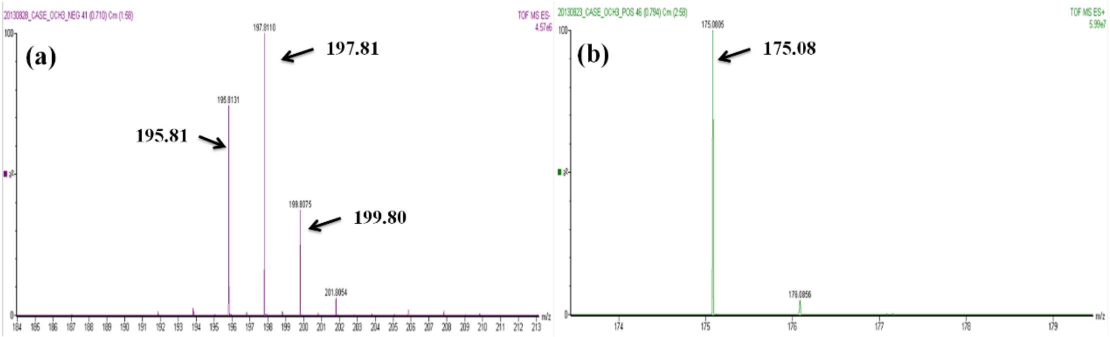

Figure S4b. Mass spectra (ionization source: electrospray ionization) of B-L MIL 5c. (**a**) Anion and (**b**) cation fragments.

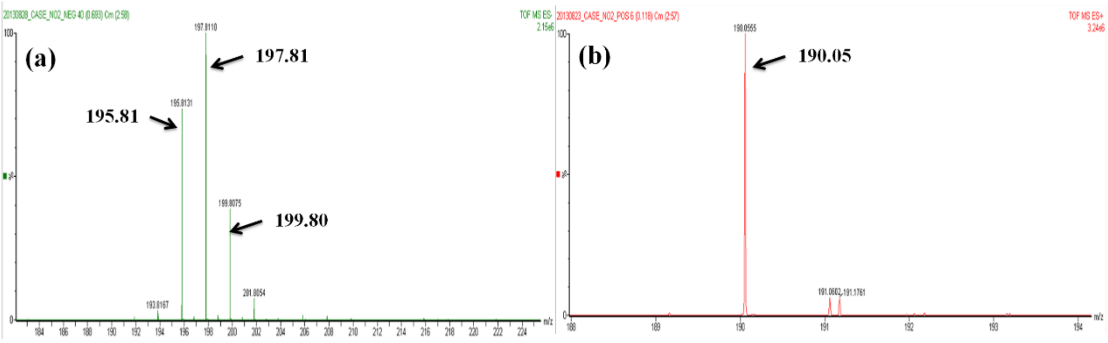

Figure S4c. Mass spectra (ionization source: electrospray ionization) of B-L MIL 5d. (**a**) Anion and (**b**) cation fragments.

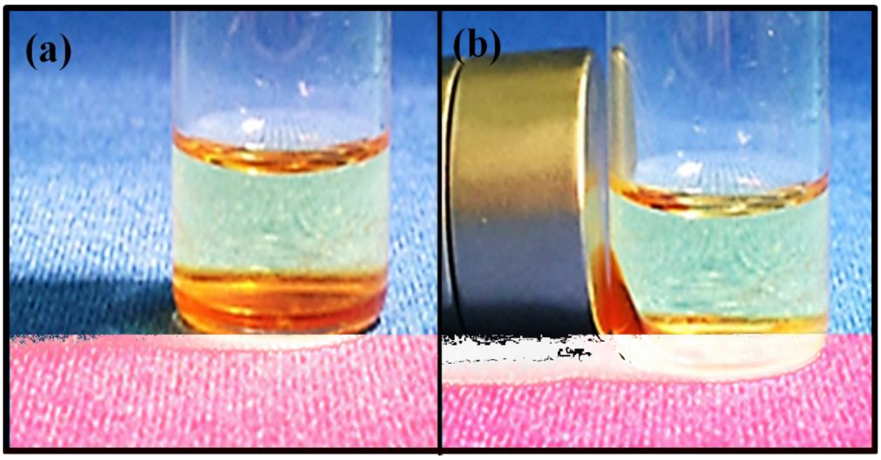

Figure S5. Photographs showing response of B-L MIL 5a to NdFeB (0.55-T) magnet. To show the displacement and distortion of 5a more clearly, ethyl ether was added to sample liquid. (**a**) Two layers of liquid, with ethyl ether at top and 5a at bottom, without magnet. (**b**) 5a attracted to magnet, becoming distorted.

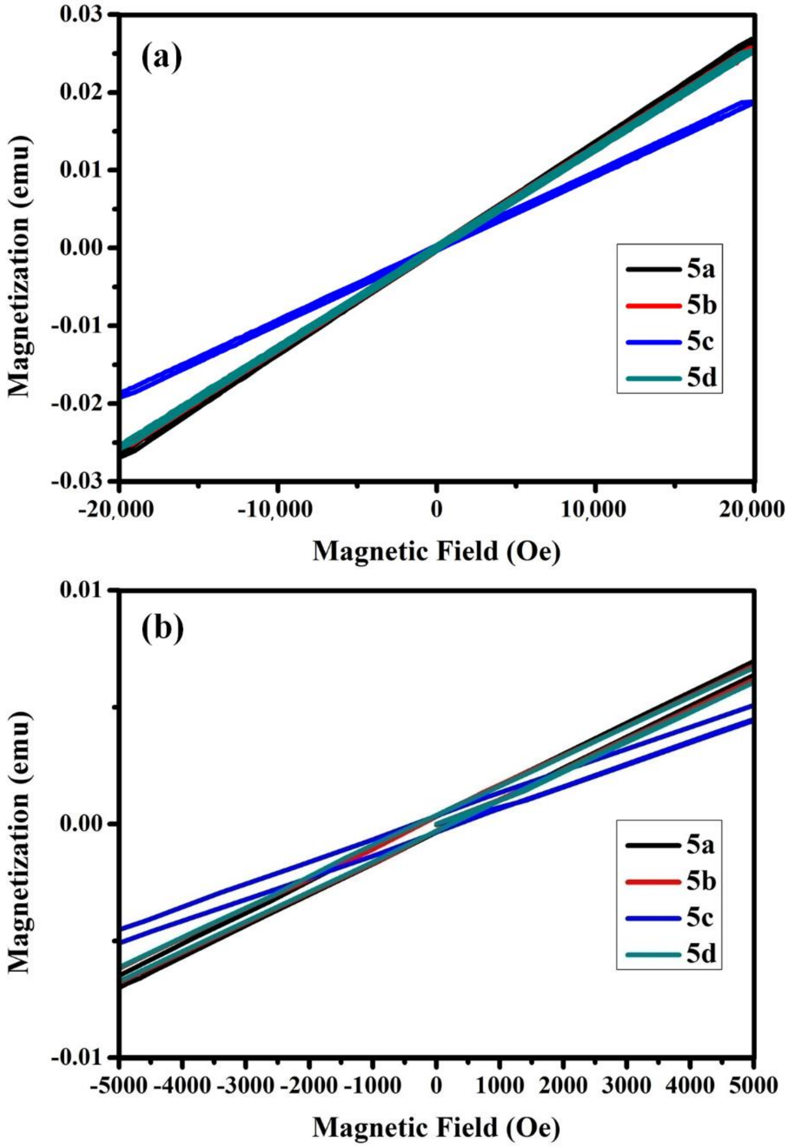

Figure S6. (**a**) Isothermal magnetization of all B-L MILs at 300 K. (**b**) enlarged view for magnetic fields of -5000 to 5000 Oe.
